# Fractal frontiers in cardiovascular magnetic resonance: towards clinical implementation

**DOI:** 10.1186/s12968-015-0179-0

**Published:** 2015-09-07

**Authors:** Gabriella Captur, Audrey L. Karperien, Chunming Li, Filip Zemrak, Catalina Tobon-Gomez, Xuexin Gao, David A. Bluemke, Perry M. Elliott, Steffen E. Petersen, James C. Moon

**Affiliations:** UCL Institute of Cardiovascular Science, University College London, Gower Street, London, WC1E 6BT UK; Division of Cardiovascular Imaging, The Heart Hospital, part of University College London NHS Foundation Trust, 16-18 Westmoreland Street, London, W1G 8PH UK; Centre for Research in Complex Systems, School of Community Health, Charles Sturt University, Albury, NSW 2640 Australia; Department of Radiology, University of Pennsylvania, Philadelphia, PA USA; Cardiovascular Biomedical Research Unit, Barts and the London School of Medicine and Dentistry, Queen Mary University of London, London, UK; Division of Imaging Sciences & Biomedical Engineering, King’s College London, London, UK; Circle Cardiovascular Imaging Inc., Panarctic Plaza, Suite 250, 815 8th Avenue SW, Calgary, AB T2P 3P2 Canada; Radiology and Imaging Sciences, National Institutes of Health Clinical Center, Center Drive, Bethesda, MA USA; Barts Heart Centre, West Smithfield, London, UK

**Keywords:** Cardiovascular magnetic resonance, Segmentation, Image processing

## Abstract

Many of the structures and parameters that are detected, measured and reported in cardiovascular magnetic resonance (CMR) have at least some properties that are fractal, meaning complex and self-similar at different scales. To date however, there has been little use of fractal geometry in CMR; by comparison, many more applications of fractal analysis have been published in MR imaging of the brain.

This review explains the fundamental principles of fractal geometry, places the fractal dimension into a meaningful context within the realms of Euclidean and topological space, and defines its role in digital image processing. It summarises the basic mathematics, highlights strengths and potential limitations of its application to biomedical imaging, shows key current examples and suggests a simple route for its successful clinical implementation by the CMR community.

By simplifying some of the more abstract concepts of deterministic fractals, this review invites CMR scientists (clinicians, technologists, physicists) to experiment with fractal analysis as a means of developing the next generation of intelligent quantitative cardiac imaging tools.

## Fractals-irregularity and complexity in nature

The earliest formal references to fractal geometry were made by Leibniz [1] in the mid-1600s. Centuries later, the first fractal prototype was abstractly introduced (only in passing) by German mathematician Georg Cantor in 1883. But the word ‘fractal’ did not come into existence until at least one century later. Inspired by the Latin *fractus*, meaning “broken”, the term was first coined in 1975 by Benoit Mandelbrot [1] to describe complex patterns that were self-similar across infinite scales. A fractal object is defined as a rough, fragmented, or detailed geometric shape that can be subdivided into parts, each of which is a reduced copy or approximate copy of the whole, where their self-similarity may be exact, quasi, or statistical.

Theoretical mathematical fractals are indeed infinitely self-similar. We can generate limited practical graphical representations of them by repeating a pattern at different scales in a recursive or iterative loop or by recursion of algebraic equations. Algebraic fractals typically require thousands or millions of iterations before their fractal nature is realised, and thus are usually visualised using computer software. Not surprisingly, widespread appreciation of fractal complexity developed only after the advent of the computer in the 1980s and thanks to Mandelbrot’s work [1].

**Fig. 1:**
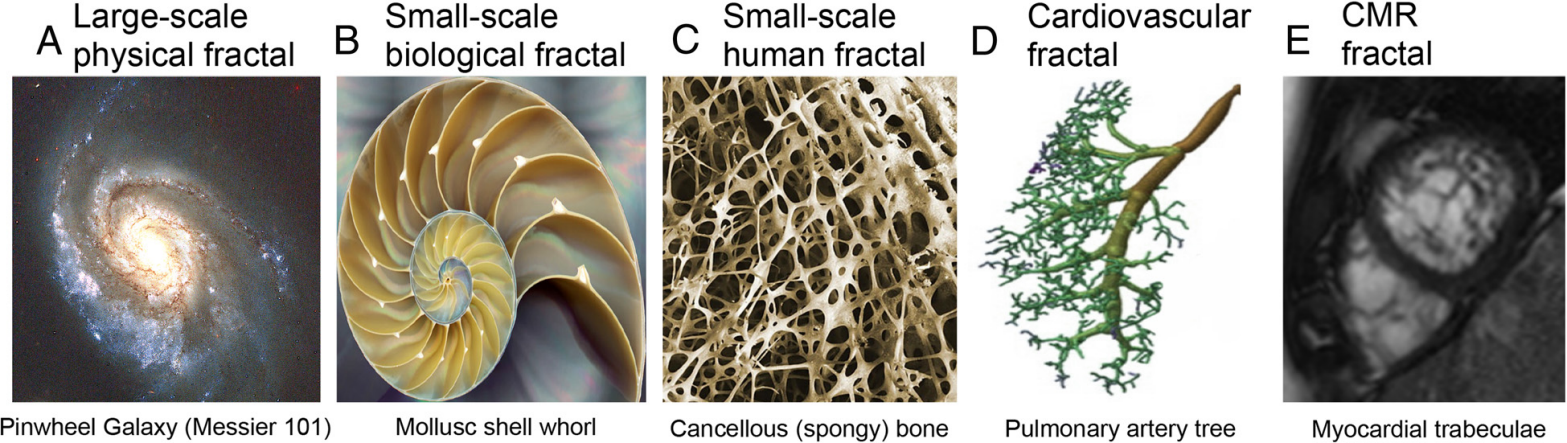
Exact self-similar elements cannot always be recognized in naturally occurring fractals. The spiral galaxy (**a**) is an example of a large-scale fractal in the physical world. Biology is full of fractal objects: from the whorls of molluscs (**b**), to the woven lattice of human cancellous bone (**c**); from the branching pulmonary arterial tree (**d**) to the trabeculated apex of the left ventricle (**e**). CMR = cardiovascular magnetic resonance

Natural quasi fractal objects, unlike theoretical fractals but much like graphical representations of fractals, are scale invariant across only a limited range of scales. We are surrounded by natural objects that iterate, branch or spiral, spanning a wide range of scales. Some large-scale examples in the physical world include recursing coastlines, branching tree and river networks, and spiralling galaxies (Fig. 1a) and hurricanes. Some small-scale examples in biology include the spirals of a nautilus and whorls of a seashell (Fig. 1b). Small-scale examples in the human body include the lattices of cancellous bone (Fig. 1c), neuronal dendrites, tumor growth patterns, and specifically for the cardiovascular system, branching vascular networks (Fig. 1d), endocardial trabeculae, and the quasi-fractal ordering of collagen and fibrosis in the diseased myocardium as seen by micro-histology [2].

**Fig. 2:**
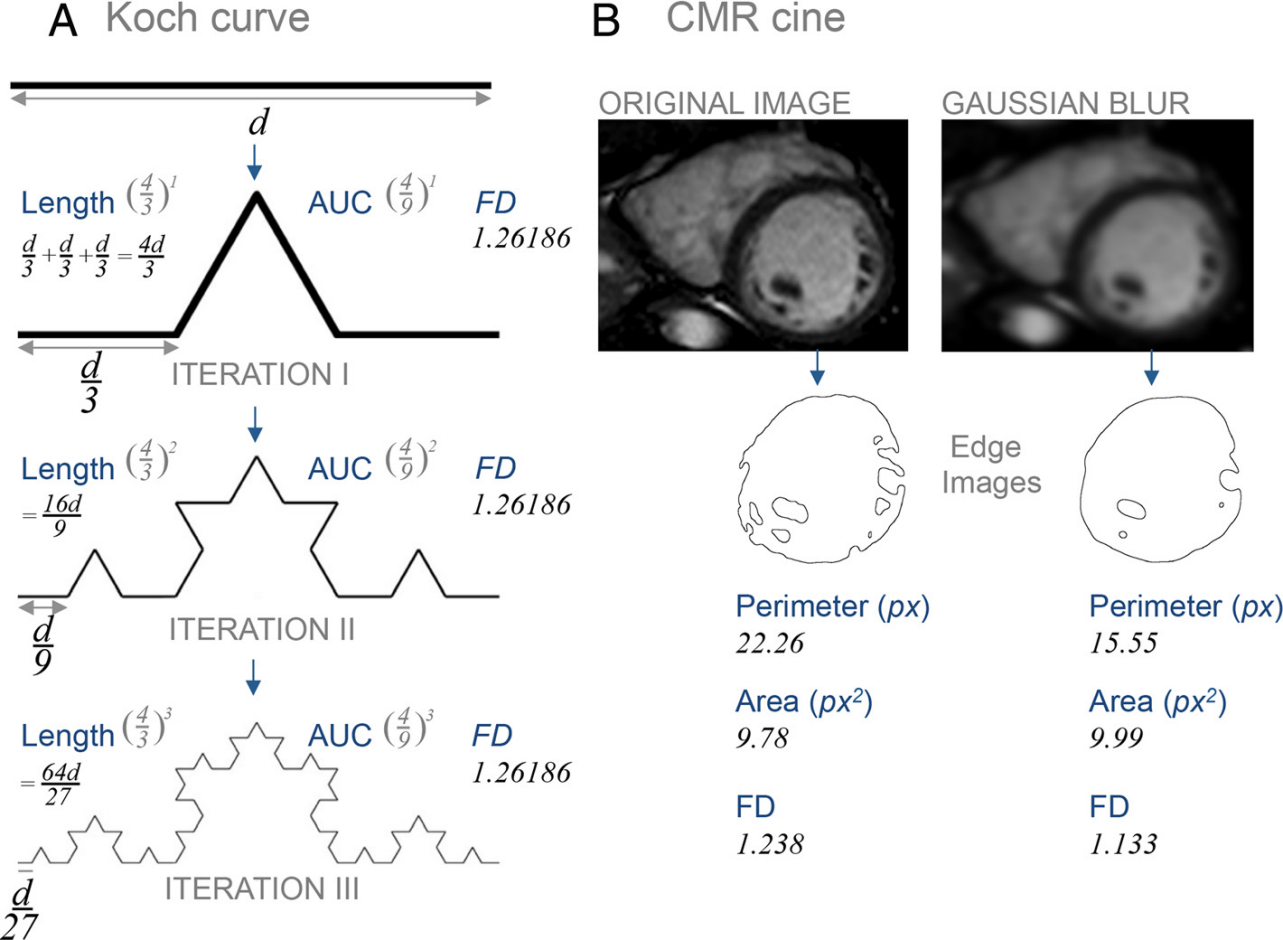
**a** The first 3 iterations of the Koch coastline, an exact geometrical fractal. It can be quantified by its perimeter, its AUC or its *FD*. With each successive iteration of the Koch coastline the original pattern is repeated at a finer level, corresponding to how with increasingly greater magnification increasingly fine detail is revealed in fractals. By traditional methods, the AUC will converge on $\frac{8}{5}$ and the perimeter of the curve after *n* iterations will be $\left(\frac{4}{3}\right)n$ times the original perimeter (4 times more lines, $\frac{1}{3}$ greater length per iteration), and since $\left|\frac{4}{3}\right|>1,$ perimeter will tend to infinity. These exemplify the inherent problem with traditional mathematics: it is capable of providing only scale-dependent descriptors that give limited insight into the motif’s overarching complexity. The *FD* of the Koch curve, on the other hand, summarises its complexity independently of scale. At every iteration (from 1 to infinity) the *FD* is invariant at $\frac{ \log 4}{ \log 3}\approx 1.26186$. Biological quasi-fractals are measured by ‘sampling’ them with an imaging ‘camera’ relevant to a particular imaging modality. Different cameras have different resolutions, but in all cases increasing resolution is similar to accumulating iterations on a mathematical fractal. Natural quasi-fractals are self-similar across a finite number of scales only—a lower limit of representation is imposed by the limit of the screen (pixel resolution). For CMR cines, blurring (quite extreme in **b**) has the same effect as setting a lower resolution for the particular sequence, and this is equivalent to having fewer fractal iterations. With such manipulation, it can be seen that the area of the set changes little (here by 2 %), the perimeter a lot (by 43 %) and the *FD* less (by 8 %). This implies that high image resolution (and a fractal approach) may not add much value when attempting to measure the left ventricular volume; but image resolution (and a fractal approach) will make a considerable difference when intricate features like trabeculae are the features of interest: the perimeter length or other 1D approach will be less robust than the *FD*. AUC = area under the curve; *d* = length of segment; 1D = one-dimension/al; *FD* = fractal dimension; px = pixels. Other abbreviation as in Fig. 1

In cardiovascular magnetic resonance (CMR), much of what we see, report, measure and compute in everyday clinical practice also has some quasi-fractal property and is amenable to description and quantification by fractal mathematics, generating an index of their space-filling. To date however, much more emphasis on Fourier analysis and processing of CMR data has existed. Fractal analysis of magnitude images is a more recent application—although more than 100 [3–6] publications indexed in PubMed have described fractal analysis in magnetic resonance imaging of the brain, only 4 publications exist for CMR [7–10]. Summing up this biological complexity in medical images is clinically important, to guide treatment decisions and improve disease diagnosis, but attempting to do so using traditional mathematics (perimeter estimates or area under the curve) is unsatisfactory—it will tend to either oversimplify the motif’s detail and/or vary with the iteration being interrogated (Fig. 2). In general, the fractal approach is ideal for measuring complicated image details that are beyond simple calliper measurement, and permits results from different scanners to be meaningfully compared.

By summarising some of the fundamental principles underpinning the science of deterministic fractals, and by pointing to existing tools and approaches, this paper invites CMR scientists to experiment with fractal analysis as a means of developing an alternative breed of quantitative cardiac imaging tools.

### How to measure

**Fig. 3:**
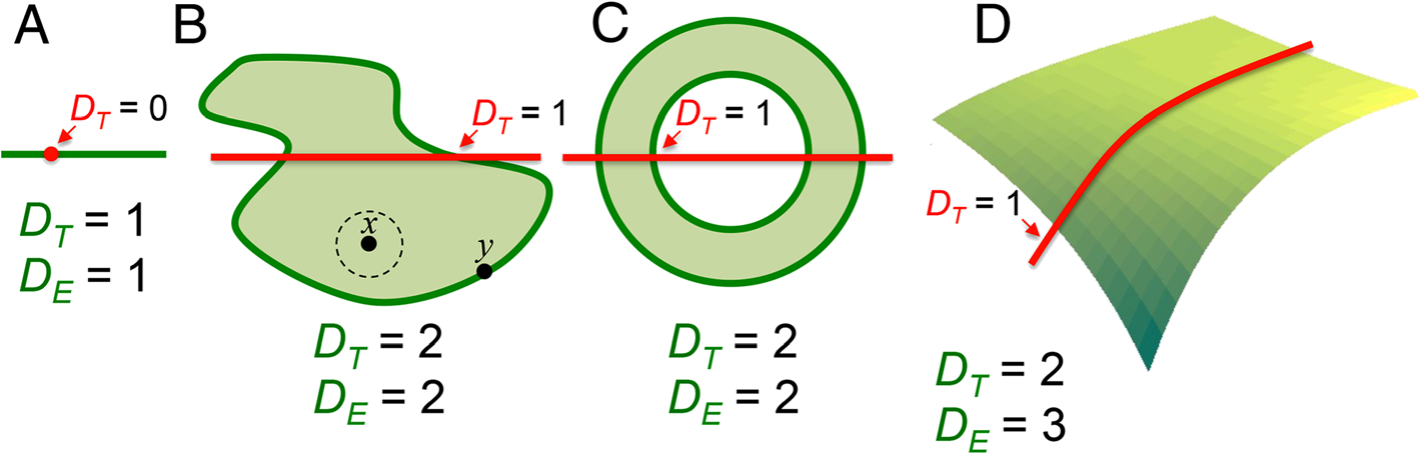
A line, square or cube all exist in Euclidean space with a certain number of dimensions described classically by *D*_*E*_ = 0 for a single point, 1 for a line (**a**), 2 for a plane (**b**) and 3 for a 3D object (**d**) [38]. The concept of topology is rooted in the idea of connectedness among points in a set. The null (empty) set in topology (∅) has no points and its *D*_*T*_ is by definition ‘-1’. A single point or a number of points makes up a ‘countable set’. In topology, a set’s *D*_*T*_ is always 1 integer value greater than the particular *D*_*T*_ of the simplest form that can be used to ‘cut’ the set into two parts [42]. A single point or a few points (provided they are not connected) are already separated, so it takes ‘nothing’ (∅) to separate them. Thus the *D*_*T*_ of a point is 0 (−1 + 1 = 0). A line (**a**) or an open curve can be severed by the removal of a point so it has *D*_*T*_ = 1. A topological subset such as **b** can have an interior, boundary and exterior. **b** has a closed boundary of points (like *y*). When its interior is empty, **b** is referred to as a boundary set. Its interior may instead be full of points (like *x*) that are not boundary points because separating them from the exterior is a neighbourhood of other points also contained in **b**. All points of the subset that are neither interior nor boundary will form the exterior of **b**. A line of *D*_*T*_ = 1 is required to split this topological set into 2 parts, therefore the *D*_*T*_ of **b** = 2. Flat disks (**c**) have *D*_*T*_ = 2 because they can be cut by a line with a *D*_*T*_ = 1. A warped surface can be cut by a curved open line (of *D*_*T*_ = 1) so its *D*_*T*_ = 2 although its *D*_*E*_ = 3. Therefore, while lines and disks have *D*_*T*_ = *D*_*E*_, warped surfaces have *D*_*T*_ one less than *D*_*E*_. *D*_*E*_ = Euclidean dimension; *D*_*T*_ = topological dimension

Geometrically a fractal would exist in between our more familiar topological dimensions (*D*_*T*_): between the 1st and 2nd *D*_*T*_, or between the 2nd and 3rd, etc. An understanding of the concept of fractal dimensionality begins therefore with at least some understanding of *D*_*T*_ and Euclidean dimensionality (*D*_*E*_) (Fig. 3). Euclidean space refers to an object’s embedding space and encompasses dimensions that we define using Cartesian coordinates (real numbers e.g., *x, y and z*). Figure 3 explains why some objects will have *D*_*T*_ = *D*_*E*_, while others will have *D*_*T*_ < *D*_*E*_. Unlike the topological and Euclidean dimensions, the fractal dimension (*FD*) measures the detailed self-similarity of fractals—the space-filling capacity of a set of points embedded in space or its *complexity*. It is related to *D*_*E*_ and *D*_*T*_ by Eq 1:

1 $$ {D}_T\ \le\ FD\ \le\ {D}_E $$

These definitions also apply to fractal analysis in CMR. The heart itself exists in three-dimensional (3D) space, but diagnostic images provide 2D data a large part of the time, from which we extract patterns. The pattern of a drawn endocardial contour, for example on a left ventricular short axis CMR cine slice, appears more complicated than a simple curved line so its *FD* will be > 1. Because it partly but not completely ‘fills’ 2D space however its *FD* will be < 2. Therefore the range of possible *FD* s for a quasi-fractal object like the endocardial contour extracted from a CMR sequence will be consistently a value between 1 and 2.

**Fig. 4:**
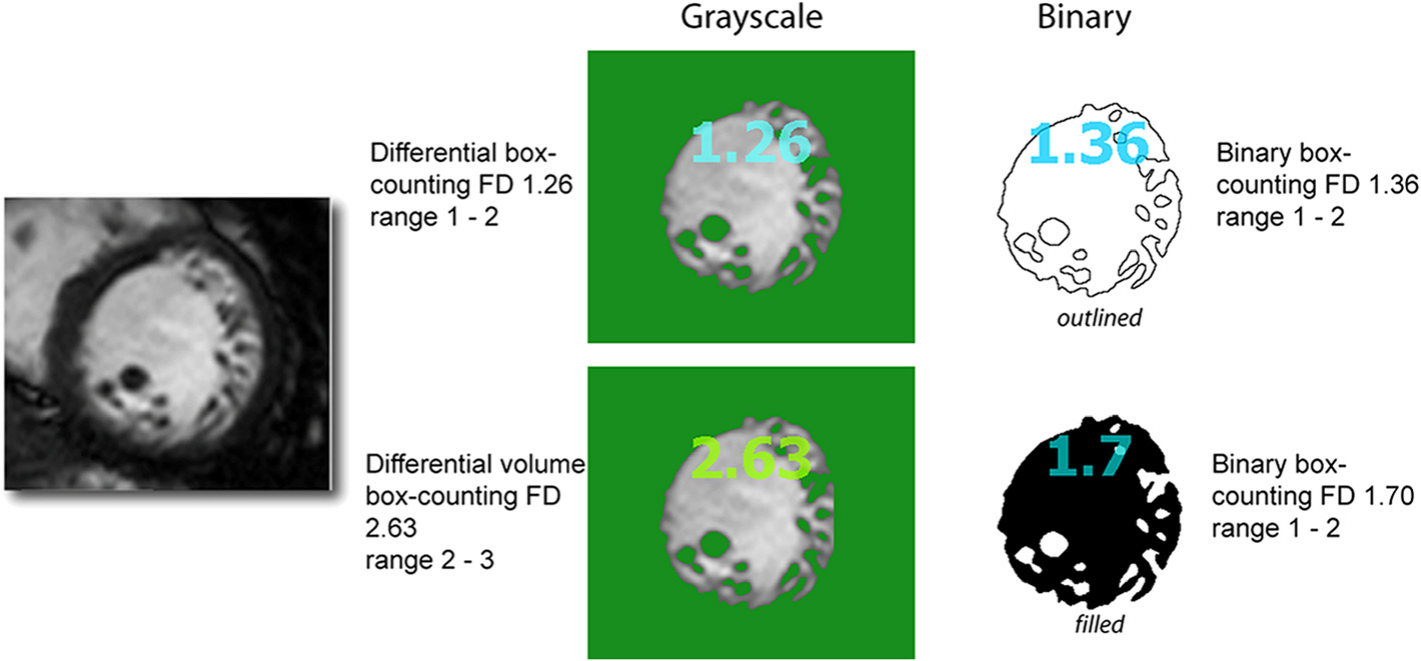
The 3D *FD* (between 2 and 3) of the grayscale cine is computed using the differential box-counting algorithm that takes 3D pixel intensity information into account. In the standard box-counting method applied to binary images as either outlines or filled silhouettes, intensity information is lost as foreground pixels are contrasted from the background pixels to derive the 2D *FD* (range 1 – 2). For the same original image and considering only the mantissa, it is usually the case that the binary *FD* is greater than the grayscale equivalent. Furthermore, the *FD* of the filled binary mask would usually be nearer to 2 when compared to the *FD* of the equivalent binary outline as the *FD* of the filled areas massively outweigh the *FD* of the edges. Abbreviations as in Fig. 2

The mathematical details of a fractal analysis are generally taken care of by software, but this is typically preceded by some medical image preparation. It may be necessary to generate the needed image format (e.g., grayscale, binary or red-green-blue (RGB) data type) or to remove image complexity unrelated to the feature to be measured. For example, a short-axis cine slice may carry signal originating from the myocardium, blood-myocardial boundary, blood pool, and surrounding tissues, all of which are measurable, either separately or together. To be able to measure the quasi-fractal properties of an endocardial contour (the blood-myocardial boundary) some image transformation would be needed in order to extract its relevant pattern, in particular its binary outline. In a segmented image, derived according to a fixed thresholding rule, the meaning of each single pixel is reduced to the binary logic of existence (pixel present/foreground) and nonexistence (pixel absent/background). Typically, the *FD* of a binary filled object (e.g., the binary mask of the blood pool) is greater than that of its binary outlined counterpart (e.g., the edge image of the endocardial contour), and the *FD* of such binary images (whether filled or outlined) will be generally greater than the equivalent *FD* [11] of the original grayscale object (Fig. 4) [12].

Assuming the preprocessing approaches used (threshold, subtract background, dilate, trace, find edges, binarise or skeletonize, either automatically or manually) are appropriate for the type of image [13, 14], then it is reasonable to expect that the *FD* of the resultant region of interest (ROI) will closely approximate the real *FD* of the aspect of the physical object or process being investigated, at least over a range of image resolutions, and that it will encode potentially valuable biological information.

**Fig. 5:**
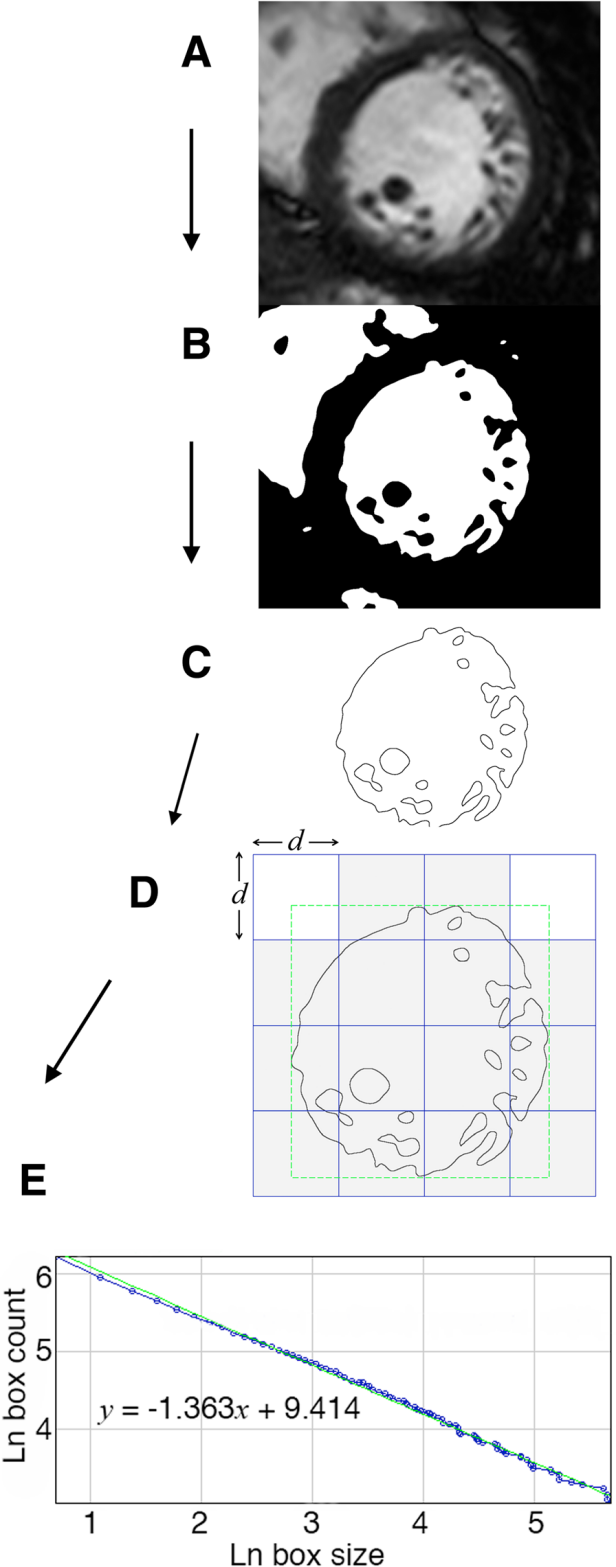
Applying fractal analysis to a 2D cine CMR slice (**a**) at the mid-left ventricular level [9]. Trabecular detail is extracted by a region-based level-set segmentation [40], followed by binarisation (**b**) and edge-detection (**c**). Binarisation eliminates pixel detail originating from the blood pool. The edge image is covered by a series of grids (**d**). The total number of sized *d* boxes making up this exemplar grid is 16, and the number of boxes *N(d)* required to completely cover the contour, 14 (2 boxes overlie blank space). For this set, box-counting will involve the application of 86 grid sizes. The minimum size is set to 2 pixels. The maximum size of the grid series is dictated by the dimensions of the bounding box (discontinuous red line) where ‘bounding box’ refers to the smallest rectangle that encloses the foreground pixels. The box diameter for each successive grid is set to drop by *d*-1 pixels each time. Through the implementation of this 2D box-counting approach, a fractal output of between 1 and 2 is expected. The log-lot plot (**e**) produces a good fit using linear regression and yields a gradient equivalent to - *FD* (1.363). *d* = box dimension; Ln = natural logarithm*; N(d) =* number of boxes. Other abbreviations as in Figs. 1 and 2

Once the ROI is extracted, the *FD* can be calculated using many analysis methods (Table 1). Each will compute a different type of *FD* but fundamentally they all measure the same property of the ROI—they are all meters of complexity. Even for a single method (e.g., box-counting) multiple algorithmic variants may exist (box-counting may use either a conventional, overlapping, folded or symmetric surface scanning approach [15]). The conventional procedure for box-counting (Fig. 5) rests on simple arbitrary scaling and can be applied to structures lacking strictly self-similar patterns. It works by systematically laying a series of grids of boxes of decreasing calibre onto the ROI and counting (at each level) the number of boxes that overlies pixel detail. The *FD* is derived from the slope of the logarithmic regression line graphing the relationship of box count and scale. The number of data points used to generate these log-log plots is related to the number of measuring steps. Theoretically, given *a priori* knowledge of the scaling rules, a mathematical fractal would generate data points that lie along a perfect straight line. The point of practical analysis, however, is to find the scaling rule in the first place. For anisotropic biological objects (like left ventricular endocardial contours) as well as for precisely generated fractal images analysed without knowledge of the scaling rule, the data points do not generally lie on a straight line, reflecting sampling limitations as well as limited self-similarity [16], thus the slope is estimated from the regression line for the log-log plot. The choice of image preparation routine and the details of the method used to gather the data for fractal analysis are important as they can either increase or decrease the correlation coefficient of the double logarithmic plot (more linear or more sigmoid fit respectively).

**Table 1:** List of fractal dimensions that are most commonly used

Dimension *Synonym*	Symbol	Context	Author, *Year described*
Fractal	*D*	Generic term first introduced by Mandelbrot	Mandelbrot, *1983*
Hausdorff *Hausdorff-Beisicovitch*	*D*_*H*_	Uses image coverage by a number of countable spheres; widely used in pure mathematics but less suitable for use with natural fractals	Hausdorff, *1919*
			Beisicovitch, *1935*
			Mandelbrot, *1983*
			Falconer, *1990*
			Gulick, *1992*
Minkowski-Bouligand *Kolmogorov*	*D*_*M*_	Uses circle sweep like for *D*_*H*_; easier to evaluate than *D*_*H*_ *;* outputs usually greater than or equal to *D*_*H*_	Mandelbrot, *1983*
			Smith, *1989*
			Schroeder, *1991*
Calliper *Perimeter-stepping, Divider, Richardson, Compass*	*D*_*C*_	Calculates the fractal complexity of a simple continuous perimeter	Richardson, *1961*
			Mandelbrot, *1967/83*
			Falconer, *1990*
			Smith, *1989*
			Peitgen, *1992*
Box-counting *Capacity*	*D*_*B*_	Uses a grid method to measure the fractal complexity of 2D and 3D noncontiguous outlines commonly encountered in biological structures	Mandelbrot, *1983*
			Falconer, *1990*
			Gulick, *1992*
			Peitgen, *1992*
Mass-radius	*D*_*MR*_	Typically used in the context of clusters and networks; can be applied to surfaces and biological objects	Caserta, *1990*
			Jelinek, *1998*
Lyapunov	*D*_*L*_	Used for measuring the dimension of strange attractors in time series analysis.	Gulick, *1992*
Packing	*D*_*P*_	Uses dense packing by disjoint balls of differing small radii.	Falconer, *1990*
Local connected set	*D*_*LC*_	Variant of box-counting applied to binary images where they are sampled pixel by pixel according to the local connectedness of each pixel	Landini, *1995*
Packing	*D*_*P*_	Uses dense packing by disjoint balls of differing small radii.	Falconer, *1990*
Grayscale box-counting *Differential box-counting Fourier Higuchi’s*	*D*_*BC*_	Does not require image segmentation; suitable for being performed in an unsupervised manner and most amenable to automation.	Sarkar, *1994*
			Azemin, *2011*
			Higuchi, *1988*

**Fig. 6:**
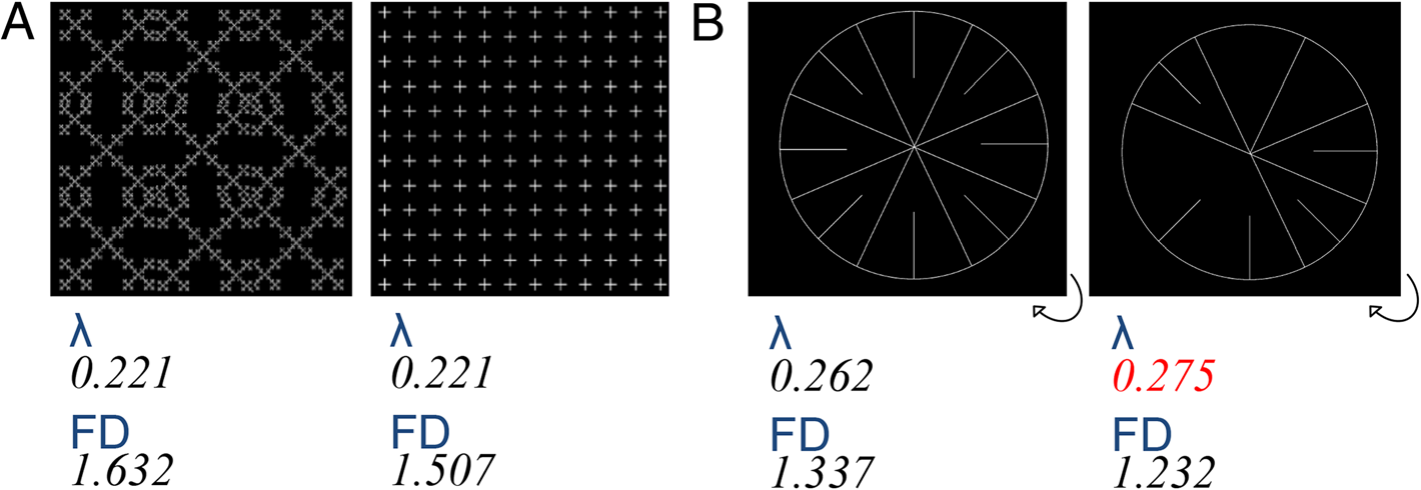
It is possible to construct a family of fractals that share the same *FD*, but differ sharply in their overall texture so they have uncorrelated values for *λ* —likewise two objects may have the same *λ* but very different *FD*. In **a**, two 2D binary sets are presented that share the same *λ* but have different *FD*. For quantifying myocardial trabecular complexity in CMR cines, *FD* was chosen over *λ* for a number of reasons: 1) experiments on grayscale short-axis imaging sequences showed *λ* was confounded by signal from the central blood pool; 2) as *λ* measures translational invariance (imagine the binary edge-image rotated clockwise as per curved arrow in **b**), it is theoretically possible for a heavily but symmetrically trabeculated heart (**b**, left image) to have a lower value for *λ* than one with fewer, more irregularly spaced trabeculae (**b**, right image). On the contrary, if there are more trabeculae, whether regularly or irregularly spaced, *FD* will always be higher. As the sole objective of this tool was to quantify trabeculae, the extra information on spatial heterogeneity encoded in *λ* could only have distracted from the biological signal of interest; 3) *λ* is a very scale-dependent meter and potentially more susceptible to differences in image resolution across vendors and CMR centres compared to *FD. λ* = lacunarity. Other abbreviations as in Figs. 2 and 3

The *FD* is not the only tool available in fractal geometry—others such as lacunarity also exist that provide a different layer of information relating more to the texture of objects [17]. Lacunarity (*λ*) mesures the size distribution of gaps (lacunae) in an image, providing a measure of heterogeneity [18]. It is the counterpart to the *FD* but the two are non-identical (Fig. 6). If an image has few, small, and regular gaps and is translationally and rotationally invariant, it will have low *λ*; if it has many large and irregular gaps with notable translational and rotational variance, it will have high *λ*. The translational invariance (spatial heterogeneity [19]) that is measured by lacunarity implies that: 1) *λ* is highly scale-dependent, meaning an image that appears highly heterogenous at low scale may appear much more homogenous at large scale producing two very different values of *λ*; and 2) *λ* (like the related box-counting fractal analysis) may be used to study non-fractal objects. *λ* and the *FD* are usually used complementarily, but for some biomedical applications lacunarity may be preferred (e.g., quantification of trabecular bone by MR [20] where the widely varying pattern of emptiness between spicules is the feature of interest, Fig. 1c), and in others the *FD* is preferred (e.g., endocardial contours with large central emptiness and edge detail, Fig. 5).

### Previous use of fractal analysis in medicine

Fractal geometry has already found effective research application in the medical imaging field across several modalities (such as plain radiography, retinal photography, ultrasonography [21], computed tomography, MR and nuclear MR [22]). It has been used to study a wide variety of processes: the complex geometries of biological cell types [23]; tumor growth patterns [24]; gene expression [25]; retinopathy [26]; cellular differentiation in space and time [27]; bone and dental matrix composition [24, 28]; brain matter changes [29] etc. Fractal methods are popular and convenient because they lend themselves to automated computer-assisted image processing providing a precise and quantitative metric. Robust measurement of biological complexity in the medical imaging field is clinically important and worth pursuing because fractal indices have been shown to permit early diagnosis of disease (in osteoporosis [20]), predict likelihood of malignancy (in mediastinal nodes imaged by endobronchial ultrasound [21]), predict outcome (of lacunar strokes on the basis of retinal vessel complexity [26]) and measure treatment response (to radiochemotherapy in malignant head and neck tumors [30]).

### Utility in CMR

Pertinent to CMR, and for certain applications (e.g., myocardial trabecular quantification), there are clear advantages in using the *FD*: because it is less susceptible to magnification, it works on different CMR sequences, with different voxel sizes acquired on different platforms; because it is independent of the size of the ROI, it works for small as well as large hearts. There are also potential limitations. For cine imaging, loss of image detail is a particular concern due to partial volume effects at the blood-myocardial boundary in the relatively extended through-plane voxel dimension. Left ventricular cine stacks may be prone to variable spatial resolution but we have previously shown how *FD* is robust to small changes in slice thickness (6 mm vs. 7 mm vs. 8 mm [10]). Future work should explore whether the higher spatial resolution of computerised tomography provides more suitable image data for fractal analysis than does CMR, especially with respect to vascular trees and probably also myocardial trabeculae provided blood-myocardial contrast is sufficient.

**Fig. 7:**
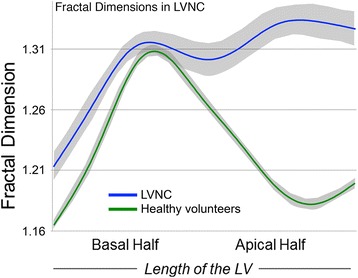
Clinical application of a fractal analysis for trabecular quantification by CMR in LVNC. It is noteworthy how in healthy hearts, it is the mid-LV that holds the greatest fractal complexity (papillary muscles), a fact that is commonly overlooked as the more intricately trabeculated apex commonly distracts. LVNC = left ventricular noncompaction. Authorization for this adaptation has been obtained both from the owner of the copyright in the original work [8] and from the owner of copyright in the translation or adaptation (*JCMR*)

Experimenting with fractal analysis of images in the CMR domain, typically involves the in-house development of scripts written for a specific programming environment (e.g., MATLAB, ImageJ [31], Insight Toolkit [ITK] [32], etc.). It may be possible to repurpose already available tools in the form of commercial and open-source fractal plugins and codes [33]. Examples include: Fractalyse (ThèMA, F), Whinrhizo (Regent Instruments Inc.), Image Pro Plus (Media Cybernetics), FDSURFFT (in MATLAB) and Fraclac for ImageJ [34]. Our group started off with Fraclac and then moved to an in-house MATLAB implementation. We applied fractal analysis to CMR cine data for trabecular quantification. In left ventricular noncompaction (*n* = 30) compared to healthy volunteers (*n* = 75) fractal analysis (Fig. 7) revealed *FD* elevation in the apical half of the left ventricle [8] (1.392 ± 0.010 versus 1.235 ± 0.004). When we studied patients at our centre with hypertrophic cardiomyopathy (*n* = 107), fractal analysis showed abnormally increased apical *FD* not only in overt disease, but also in sarcomere gene mutation carriers without left ventricular hypertrophy (G + LVH-, 1.249 ± 0.07) compared to controls (1.199 ± 0.05) [9]. In a multi-centre setting high *FD* was further shown to predict hypertrophic cardiomyopathy sarcomere gene mutation carriage in G + LVH- (*n* = 73) [10]. Applied to 2547 participants in the population-representative MESA study, fractal analysis was able to provide ethnically-appropriate normal reference ranges for left ventricular endocardial complexity [35].

Whether to measure endocardial complexity or any other imaging feature of interest, all novel CMR fractal tests will invariably need to satisfy the usual STAndards for the Reporting of Diagnostic accuracy studies (STARD) [36]. To become useful clinical tools, they will need to pass the 15 developmental “check-points” [37]. Table 2 underscores how two efforts in this field are still some way off from clinical utility (e.g., the further developed of the two is at step 11—development of normal reference values).

**Table 2:** The 15 steps needed to turn a fractal tool in a clinically valid test (also considering STARD [39] criteria)

Developmental step	Fractal quantification of trabecular complexity [9]	Fractal quantification of the spatial distribution of pulmonary flow [7]
1. Technical development and theoretical basis of the test	*Achieved –* method first implemented in Java [8] and then in MATLAB [9] to improve computational efficiency; many segmentation algorithms tested before choosing a region-based level-set function [40]	*Achieved –* fractal dimension used as an index of pulmonary perfusion heterogeneity; image preparation included a coil inhomogeneity correction
2. Comparison with gold-standard or tissue biopsy (animal models and then human biopsy material)	*Achieved –* validated against episcopic mouse embryo datasets and using synthetically constructed phantoms with well-known *FD*: 1) regular geometrical objects (plane, cube surface, sphere surface) and 2) ideal monofractal signals (4^th^, 5^th^ and 6^th^ iteration of the Sierpinski carpet or 9^th^, 10^th^ and 11^th^ iteration of the Sierpinski gasket)	*Part achieved –* validated using 3 MR reference phantoms applied to each patient’s chest
3. Detection of changes in established disease compared with normals	*Achieved – FD* in left ventricular noncompaction compared to healthy volunteers	*Not achieved*
4. Correlation with other equivalent cardiac imaging markers	*Achieved –* correlated with perimeter and with noncompacted/compacted wall thickness ratio [41]	*Achieved –* comparison is made with relative dispersion and the geometric standard deviation
5. Correlation with other relevant biomarkers	*Not achieved*	*Achieved –* data correlated with pulmonary function test from spirometry and repeated for three different inspired oxygen concentrations (normoxia, hypoxia and hyperoxia)
6. Demonstration of the test in more than one condition	*Achieved –* noncompaction and also subclinical and overt hypertrophic cardiomyopathy (and hypertension, in press)	*Not achieved*
7. Demonstration of test sensitivity (early disease or change with age)	*Achieved –* in subclinical hypertrophic cardiomyopathy	*Not achieved*
8. Demonstration of ability to track changes over time	*Not achieved*	*Not achieved*
9. Demonstration of predictive or prognostic value of the test	*Achieved –* in combination with other CMR imaging markers, high *FD* shown to predict sarcomere gene mutation carriage in subclinical hypertrophic cardiomyopathy	*Not achieved*
10. Standardization of the test (reproducibility, different equipment, in non-research settings, quality control, limitations of test)	*Achieved –* intra- and inter-observer variability, inter-scanner reproducibility, field-strength and slice-thickness independence demonstrated; community roll-out started through open-source release of an OsiriX plugin and development of an equivalent commercial version (in cvi42, Circle Cardiovascular Imaging)	*Not achieved*
11. Development of robust age/ethnic normal reference ranges	*Achieved –* through analysis of the Multi-Ethnic Study of Atherosclerosis (in press); robust to multi-centre/multi-vendor implementation	*Not achieved*
12. Changes in biomarker remain tied to the disease after treatment	*Not achieved*	*Not achieved*
13. Demonstration of test as surrogate trial end point	*Not achieved*	*Not achieved*
14. Clinical use and regulatory approval of test	*Not achieved*	*Not achieved*
15. Prove that test use improves clinical outcome	*Not achieved*	*Not achieved*

*Not achieved* marks a developmental milestone that has not yet been reached/published to our knowledge

Nonetheless, on the broader frontier, there is reason for optimism with regard to developing useful CMR applications in the future. We think that potential, as yet untested applications could include such things as textural analysis to quantify scar in late gadolinium enhancement images; spatiotemporal analysis to track cardiac motion of cine objects; stochastic fractal models [38] to study nonperiodic fluctuations in physiological parameters in MR flow data; and fractal analysis in general to aid pattern recognition in pixel-wise parametric mapping.

## Conclusions

Although the description of modern fractal analysis by Mandelbrot occurred more than 40 years ago and in spite of clinical practice bringing us face to face with multifarious fractal features daily, the CMR community is only beginning to evaluate potential applications of fractal analysis to cardiac imaging. This review reminds us of the accessibility of fractal mathematics and methods and aspires to attract more cardiac imagers to the library of efficient fractal analysis tools available, as well as invite them to innovate. A deeper fractal exploration of the human heart by CMR has the ability to teach us new facts relating to cardiac function, haemodynamics and tissue characterisation. With additional validation, software tools based on fractal analysis may ultimately prove to have clinical utility in the field of CMR.

## Abbreviations

CMR: Cardiovascular magnetic resonance
2/3D: Three-dimensional
*D*_*T*_: Topological dimension
*D*_*E*_: Euclidean dimension
*FD*: Fractal dimension
G+LVH-: Sarcomere gene mutation carriers without left ventricular hypertrophy
*λ*: Lacunarity
ROI: Region of interest
